# SCARF Genes in COVID-19 and Kidney Disease: A Path to Comorbidity-Specific Therapies

**DOI:** 10.3390/ijms242216078

**Published:** 2023-11-08

**Authors:** Sol Carriazo, Daria Abasheva, Deborah Duarte, Alberto Ortiz, Maria Dolores Sanchez-Niño

**Affiliations:** 1 Division of Nephrology, Department of Medicine, University Health Network, University of Toronto, Toronto, ON M5G 2C4, Canada; somacaju@hotmail.com; 2 RICORS2040, 28049 Madrid, Spain; aortiz@fjd.es; 3 Department of Nephrology and Hypertension, IIS-Fundacion Jimenez Diaz UAM, 28049 Madrid, Spain; daria.abasheva@quironsalud.es (D.A.); deborah.duarte@quironsalud.es (D.D.); 4 Departamento de Medicina, Facultad de Medicina, Universidad Autónoma de Madrid, 28049 Madrid, Spain; 5 Departamento de Farmacología, Facultad de Medicina, Universidad Autónoma de Madrid, 28049 Madrid, Spain

**Keywords:** acute kidney injury, chronic kidney disease, SCARF, COVID-19, genetic predisposition, mortality

## Abstract

Severe acute respiratory syndrome coronavirus-2 (SARS-CoV-2) causes coronavirus disease 2019 (COVID-19), which has killed ~7 million persons worldwide. Chronic kidney disease (CKD) is the most common risk factor for severe COVID-19 and one that most increases the risk of COVID-19-related death. Moreover, CKD increases the risk of acute kidney injury (AKI), and COVID-19 patients with AKI are at an increased risk of death. However, the molecular basis underlying this risk has not been well characterized. CKD patients are at increased risk of death from multiple infections, to which immune deficiency in non-specific host defenses may contribute. However, COVID-19-associated AKI has specific molecular features and CKD modulates the local (kidney) and systemic (lung, aorta) expression of host genes encoding coronavirus-associated receptors and factors (SCARFs), which SARS-CoV-2 hijacks to enter cells and replicate. We review the interaction between kidney disease and COVID-19, including the over 200 host genes that may influence the severity of COVID-19, and provide evidence suggesting that kidney disease may modulate the expression of SCARF genes and other key host genes involved in an effective adaptive defense against coronaviruses. Given the poor response of certain CKD populations (e.g., kidney transplant recipients) to SARS-CoV-2 vaccines and their suboptimal outcomes when infected, we propose a research agenda focusing on CKD to develop the concept of comorbidity-specific targeted therapeutic approaches to SARS-CoV-2 infection or to future coronavirus infections.

## 1. Introduction

**Figure 1:**
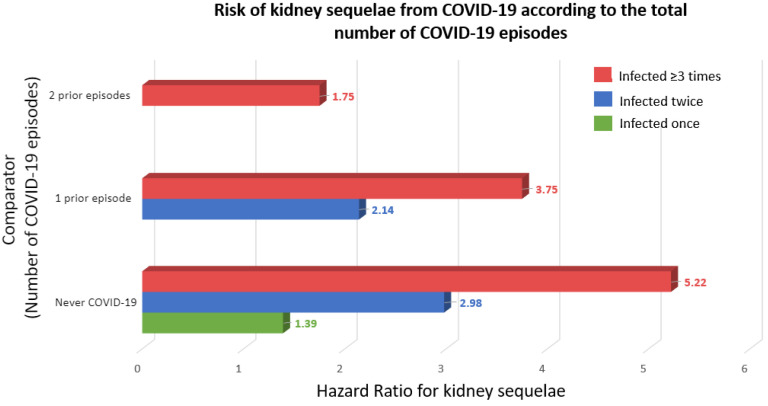
Risk of kidney sequelae from COVID-19 according to the total number of COVID-19 episodes [3]. Kidney sequelae were defined as acute kidney injury or chronic kidney disease. Outcomes were defined at time of first incidence of their component individual sequela.

Severe acute respiratory syndrome coronavirus 2 (SARS-CoV-2) has caused over 769 million confirmed cases of coronavirus disease 2019 (COVID-19) and over 6.95 million deaths worldwide, as reported by the World Health Organization (WHO), but the real numbers are believed to be much higher [1]. The WHO declared the COVID-19 outbreak a pandemic on 11 March 2020, and on 5 May 2023, declared that it is no longer a public health emergency of international concern [2]. The disease is now considered endemic. In this regard, it is still topical to advance our understanding of the pathogenesis of COVID-19 and the molecular mechanisms that determine the susceptibility to severe disease. Thus, the peak number of globally diagnosed cases was observed as recently as December 2022, and an uptick of cases driven by novel varieties was observed in July–August 2023 [1]. Additionally, any acquired knowledge will be helpful in managing the next viral pandemic outbreak. Moreover, reinfection contributed to additional risks of death (HR: 2.17, 95% CI 1.93–2.45), hospitalization (HR = 3.32, 95% CI 3.13–3.51), and sequelae among different organ systems, compared with no reinfection, despite vaccination status [3]. Kidney disorders showed the highest risk after reinfection (HR = 3.55, 95% CI = 3.18–3.97; burden = 38.31, 95% CI = 32.86–44.37), and this risk remained elevated independently of the status of vaccination [3] (Figure 1).

A plethora of manuscripts have identified host genes hijacked by SARS-CoV-2 and have explored the genetic predisposition to COVID-19 severity, and they have improved our understanding of the role of host genes in facilitating or limiting disease severity [4,5]. Key host genes include those encoding **S**ARS-CoV-2 and **c**oronavirus-**a**ssociated **r**eceptors and factors (SCARFs), that is, molecules involved in SARS-CoV-2 cell entry, replication, and assembly, as well as those encoding host defense mechanisms. In a recent example, a common HLA-B allele (HLA-B*15:0) explains why around 20% of persons infected by SARS-CoV-2 remain asymptomatic, as it is associated with memory T-cell cross-reactivity to a peptide from seasonal coronaviruses [6]. However, an understanding of the molecular mechanisms of susceptibility to severe COVID-19 in patients with kidney disease has lagged, despite chronic kidney disease (CKD) being the most common risk factor for severe COVID-19 worldwide and one resulting in the most increase in the risk of COVID-19-related death [7,8,9]. We now first review the concepts of CKD and acute kidney injury (AKI) and the clinical interaction between COVID-19 and CKD or AKI. This is followed by an assessment of persistent unmet needs, ranging from the suboptimal response to SARS-CoV-2 vaccines in some populations of patients with CKD to the persistent risks represented by novel coronaviruses or SARS-CoV-2 variants. We then proceed to assess and extract data regarding the recent information on the specific molecular pathogenesis of COVID-19-associated AKI and the molecular mechanisms that may underlie the increased severity of COVID-19 in patients with CKD, which illustrate the feasibility and need to better understand the molecular pathogenesis of COVID-19 in the context of kidney disease as a means to develop kidney disease-specific targeted therapeutic approaches that improve the COVID-19 outcomes in patients with kidney disease. Finally, we propose a research agenda.

## 2. Acute Kidney Injury and Chronic Kidney Disease

**Figure 2:**
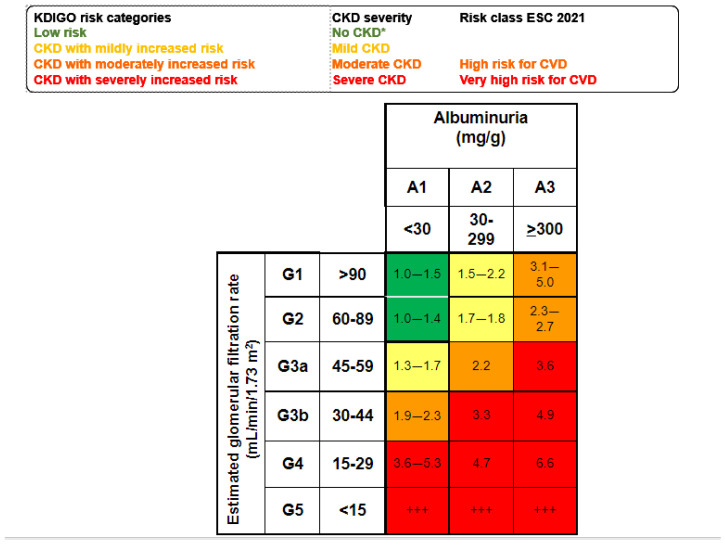
Mild, moderate, and severe CKD according to KDIGO risk categories based on simultaneous assessment of eGFR and albuminuria. Albuminuria is usually assessed as the urinary albumin–creatinine ratio (UACR in a spot urine sample. Figure shows the risk of all-cause death for each cell) [10,15]. * No CKD if ther is no other evidence of CKD as imaging, hematuria or others. +++ Kidney failure: patients needing kidney replacement therapy and cardiovascular risk is extremely high.

According to Kidney Disease: Improving Global Outcomes (KDIGO), kidney disease may be acute or chronic, and the diagnostic criteria are used to identify patients with adverse health outcomes [10,11]. Since 2002, CKD has been defined as the presence of structural and/or functional alterations in the kidneys persisting for longer than 3 months with an impact on health [12]. However, the current, more precise definition dates from 2012 [10,13]. This timing should be considered, as when the SARS-CoV-2 pandemic hit in 2020, most healthcare professionals worldwide had not studied the current concept of CKD in medical schools and neither had the health authorities. Consequently, the striking negative impact of COVID-19 in persons with CKD flew initially under the radar, unlike other comorbidities that were soon identified as risk factors for severe COVID-19, such as diabetes, hypertension, and cardiovascular disease [14]. The diagnostic criteria that by themselves allow for the detection of CKD include an estimated glomerular filtration rate (eGFR) < 60 mL/min/1.73 m^2^ or urinary albumin–creatinine ratio ≥ 30 mg/g, or pathological changes in the urinary sediment, kidney histology or imaging, or kidney transplantation [13]. eGFR and albuminuria allow for the categorization of CKD into mild, moderate, or severe categories, which are associated with increasing risks of CKD progression, premature all-cause death, or AKI, among others [15] (Figure 2).

Globally, there are 850 million people with CKD, and the number is expected to increase as the world population ages [16,17,18]. Prior to the SARS-CoV-2 pandemic, it was estimated that, by 2040, CKD would be the fifth global cause of death, as it is growing faster than most other common causes of death [16,17]. Indeed, the loss of key kidney functions, such as the production of the anti-aging protein Klotho, or the decrease in glomerular filtration and tubular secretion of waste molecules leading to the accumulation of uremic toxins, is believed to accelerate biological aging, leading to premature death, mainly from cardiovascular causes, infection, malignancy, and the lack of access to kidney replacement therapy (KRT) [16].

The progression of CKD is frequently non-linear, as CKD predisposes to AKI, and AKI may accelerate the progression of CKD [19,20]. As for CKD, the cut-off points selected to define AKI (an increase in serum creatinine by ≥0.3 mg/dL within 48 h) are associated with an increased risk of death that increases to 45–50% in those requiring KRT and may persist for up to a year [21,22,23].

## 3. CKD as a Risk Factor for COVID-19

Identifying the factors associated with severe COVID-19 is important to protect those in high-risk groups and, eventually, develop specific therapeutic approaches if the molecular mechanisms underlying the increased susceptibility are identified. CKD is the most common and the most influential comorbidity associated with COVID-19 severity. According to the prevalence data from the Global Burden of Diseases, Injuries, and Risk Factors Study (GBD) together with the UN population estimates for 2020, CKD is deemed the most prevalent risk factor for severe COVID-19 in adults [9]. In addition, CKD alone explains the increased risk of severe COVID-19 in approximately 5% of the global population. Another study conducted in the UK using primary care electronic records of 17,278,392 persons examined the factors associated with COVID-19-related death [8]. While older age, expectedly, showed the strongest association with poor outcomes, CKD was among the top five comorbidities conferring the highest risk of COVID-19-related death. The adjusted HR was 2.52 (2.33–2.72) for subjects with G4–5 CKD (eGFR < 30 mL/min/1.73 m^2^), 3.53 (2.77–4.49) for transplant recipients, and 3.69 (3.09–4.39) for patients with kidney failure, including those receiving dialysis. The Madrid REMER Registry study also showed that, in 2020, COVID-19 was the most common cause of death among patients on kidney replacement therapy (KRT), with overall mortality increasing by 34% compared with the average in the previous decade [24]. In this regard, patients on dialysis are at high risk of death from infection as a category encompassing different microbes. While the rate of mortality from infection decreased from 224 to 163 per 10,000 person-years for those commencing dialysis in 1980–2005 and 2006–2018, respectively, it remains over 20-fold higher than in the general population and disproportionally affects women and minorities [25]. Thus, the increased severity of COVID-19 should not come as a surprise, and the key question is to what extent the predisposition of CKD patients to severe COVID-19 is a further manifestation of the non-specific immune suppression in this population, or whether it is driven by specific factors that merit specific management.

## 4. COVID-19, AKI, and Mortality

COVID-19 may be complicated by AKI. This may result from systemic cytokine release syndrome due to the SARS-CoV-2 infection of kidney cells, specific disease entities such as collapsing focal segmental glomerulosclerosis or thrombotic microangiopathy, or a combination of these and other factors [26,27,28,29,30]. As is the case for AKI in other contexts, AKI is associated with an increased risk of death in patients with COVID-19 [31,32,33]. In the regions and countries hit earliest and hardest by the pandemic, the lack of enough resources to provide both ventilation support and KRT to all those in need may have contributed to a further increase in the mortality of COVID-19 AKI [34]. The fact that there is evidence of the infection of kidney cells during AKI and that AKI in COVID-19 is associated with adverse outcomes means that it is also of interest to understand the susceptibility of kidney cells to SARS-CoV-2 infection. Indeed, the SARS-CoV-2 infection of kidney cells was associated with an increased risk of death in patients with COVID-19 [35].

## 5. Suboptimal Response to SARS-CoV-2 Vaccines in Some Patient Populations with CKD

The most obvious preventive measure for those at high risk of severe COVID-19 is vaccination [36]. However, patients on dialysis and even more kidney transplant recipients have suboptimal responses to vaccines. As an example, patients on dialysis require specially designed regimens to optimize the response to vaccination against hepatitis B virus, and despite that, some patients are not immunized or are immunized only transiently [37]. There are multiple anti-SARS-CoV-2 vaccines, and reviewing the CKD literature on all of them is beyond the scope of this review. However, the mRNA-based vaccines (BNT162b2 and mRNA-1273) have been prospectively assessed using the same methods in patients with CKD not on dialysis, on hemodialysis/peritoneal dialysis, and those who underwent transplantation, and this may provide a flavor of the issues. After the initial vaccination schedule, the BNT162b2 (30 μcg) vaccine was associated with a six-fold higher risk for negative humoral response than mRNA-1273 (100 μcg), likely due to the different dose, as is the case for hepatitis B virus vaccines [38]. During the first year after vaccination, patients with non-dialysis CKD and those on dialysis presented good anti-spike antibody responses. However, anti-spike antibodies decreased over time. A third dose of vaccine induced seroconversion in a high percentage of antibody-negative patients after two doses, although responses were poorer in kidney transplant recipients [39]. The fourth dose seroconverted 72% of previously negative patients. Higher anti-spike antibody titers at 12 months were independently associated with repeated exposure to antigen (fourth vaccine dose and/or previous breakthrough infections). Breakthrough COVID-19 requiring admission was observed in CKD patients with lower antibody titers, and the least benefit from the fourth dose was observed in patients with the highest need for a vaccine booster (i.e., those with lower pre-booster antibody titers or kidney transplant recipients) [40]. Overall, kidney transplant recipients presented suboptimal responses after any vaccination schedule (initial, third, and fourth dose). Some patients had a persistently negative humoral response despite boosters [41], and the mortality of patients on KRT remains high despite increased access to critical care [42].

## 6. Multiple Severe Coronavirus Disease Outbreaks in the 21st Century

COVID-19 was the third major severe coronavirus outbreak in the 21st century. Although most reported cases of COVID-19 are mild, it is highly transmissible, accounting for the high global number of cases and death toll. Before the ongoing pandemic, two human coronaviruses were considered highly pathogenic. In 2003, SARS-CoV was identified as the causal agent of an atypical pneumonia disease termed SARS, affecting mainly China and Hong Kong [43]. In 2012, another novel coronavirus, now known as Middle East respiratory syndrome coronavirus (MERS-CoV), was discovered in Saudi Arabia in a patient presenting with pneumonia and multiorgan dysfunction [44]. SARS and MERS share similar clinical presentations with COVID-19, including high fever, chills, dyspnea, and dry cough, but are characterized by predominantly nosocomial transmission and higher case fatality rates [43,44]. The high prevalence of coronaviruses in animals and their frequent recombination lead to probable future spillovers to humans, causing periodic disease outbreaks [45]. Overall, the fact that there were three major zoonotic coronavirus outbreaks causing human disease over less than 20 years should be interpreted as the existence of a high risk for future novel zoonotic coronavirus outbreaks causing human disease. This risk exists on top of the risk for novel SARS-CoV-2 mutations that increase the transmissibility or severity of the disease. Both facts mean that there is a high need for a better understanding of the molecular mechanisms that underlie the increased risk of death from COVID-19 (or future novel coronaviruses) in specific populations so that comorbidity-specific preventive and/or therapeutic approaches can be developed.

Overall, the suboptimal outcomes of patients with CKD who develop COVID-19 or patients with COVID-19 who develop AKI and the persistent risks related to novel coronaviruses or SARS-CoV-2 variants highlight the need for a better understanding of the molecular pathogenesis of SARS-CoV-2 infection and its interaction with kidney cells or with the systemic consequences of CKD, with the ultimate aim of developing novel targeted therapeutic approaches.

## 7. Molecular Pathogenesis of COVID-19-Associated AKI

Recently, the gene expression of kidneys from patients with COVID-19 was characterized, providing novel insight into the pathogenesis of kidney injury and, specifically, kidney injury when kidney cells are infected with SARS-CoV-2 [35]. Interestingly, two main components drove the kidney gene expression difference between COVID-19 status and the presence of renal SARS-CoV-2 RNA. Inflammation pathways were prominent among differentially expressed genes [35]. Additionally, IFN-α and -γ response pathways were specifically enriched in SARS-CoV-2-infected kidneys. The COVID-19 SARS-CoV-2-infected kidney transcriptomic signature differed from another viral nephropathy, hantavirus nephropathy [35]. Furthermore, the kidney transcriptomic signature of COVID-19 AKI was compared with that of AKI in the absence of COVID-19. Overall, there were 1191 differentially expressed genes (DEGs) in COVID-19 AKI, but none were detected in non-COVID-19 AKI, serving as potential specific players of COVID-19-induced AKI (647 upregulated over 1.5-fold and 347 downregulated to less than 0.5-fold [35] (Table 1).

**Figure 3:**
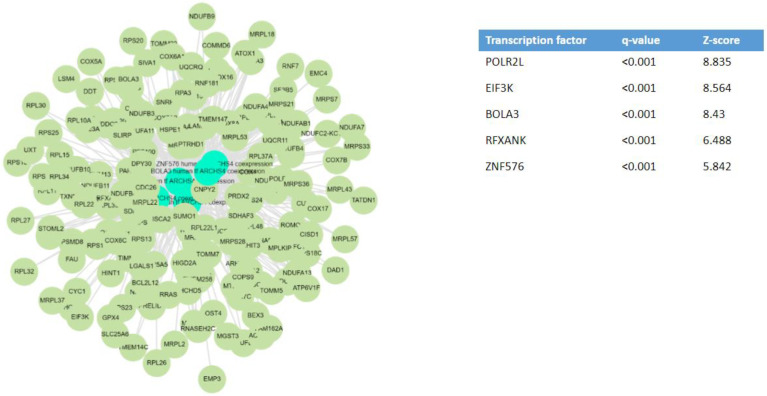
Key transcription factors that may underlie the differential gene expression observed in human COVID-19-dependent AKI [35]. Transcription factor enrichment analysis from differentially expressed genes in COVID-19 AKI, but non-differentially expressed in non-COVID-19 AKI, was performed using the bioinformatic platform Enrichr-KG [46]. NCBI GEO under the accession number GSE210622.

When analyzing the DEGs using the bioinformatic platform Enrichr-KG [46], the most relevant transcription factors potentially driving the differences in kidney gene expression between COVID-19 AKI and non-COVID-19 AKI were *POLR2L, EIF3K, BOLA3, RFXANK*, and *ZNF576* (Figure 3). *POLR2L* and *EIF3K* also contributed to explaining the full range of upregulated genes. The authors also identified an X-linked inhibitor of apoptosis-associated factor 1 (*XAF1*) as a critical target of SARS-CoV-2 infection of the kidneys [35].

## 8. SCARF Genes

**Figure 4:**
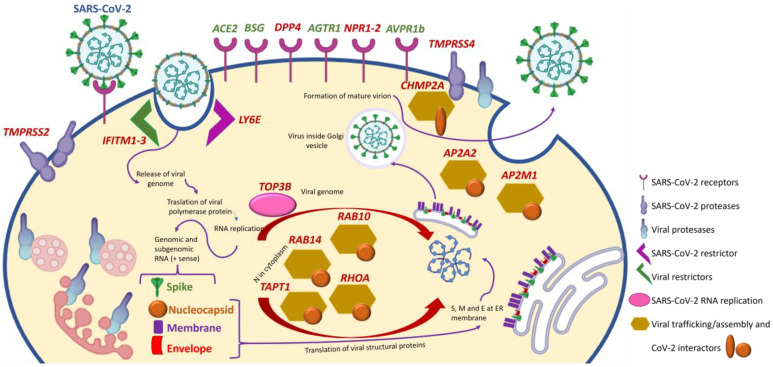
Selected SCARF genes and impact of CKD. Those differentially expressed in at least one organ (kidney, lung, heart, or aorta) of mice with CKD and whose differential expression may be expected to increase the severity of COVID-19 are identified in red. The increased expression of all the genes shown will favor the biology of SARS-CoV-2 except for *IFITM1–3* and *LY6E*, whose increased expression would impair SARS-CoV-2 entry into cells. Modified from [51] with permission.

SARS-CoV-2 and coronavirus-associated receptors and factors (SCARFs) are molecules involved in SARS-CoV-2 cell entry, replication, and assembly or that may be involved in these processes based on prior knowledge of the biology of other coronaviruses [4]. The entry mechanism of SARS-CoV-2, similar to other human coronaviruses, involves initial viral spike protein binding to a cell-surface receptor and its posterior cleavage by a host protease [46,47] (Figure 4). For both SARS-CoV and SARS-CoV-2, angiotensin-converting enzyme 2 (ACE2) in the cell membrane was identified as the primary receptor, and transmembrane serine protease type 2 (TMPRSS2) was indicated as the main cell-surface protease promoting virus entry [48]. However, the expression levels of ACE2 in the lung are relatively low and mostly limited to type 2 alveolar cells, while in other organs less affected by COVID-19, such as the small intestine, colon, or testis, ACE2 expression is much higher [49]. Thus, the evidence regarding ACE2 expression levels in different tissues and its correlation with COVID-19 clinical manifestations suggests that alternative factors are implicated in SARS-CoV-2 entry facilitation. This may include other molecules as well as the polarized distribution of ACE2. For example, proximal tubular cells express high amounts of ACE2 but on the tubular lumen [50]. Given the size of the SARS-CoV-2 virion, it is not expected to traverse a healthy glomerular filtration barrier, so proximal tubular cell entry via ACE2 would require either the disruption of the glomerular filtration barrier (e.g., glomerulopathy) or tubular injury (e.g., AKI) that disrupts the continuity of the tubular epithelial layer or alters the localization of ACE2 expression.

Single-cell RNA sequencing identified cells expressing 28 SCARF genes in healthy human tissues [4]. SCARF genes include entry factors (ACE2 and TMPRSS2) and other potential cell-surface receptors validated in human cells that may facilitate cell entry as evaluated for SARS-CoV, SARS-CoV-2, hCoV-229E or MERS-CoV, such as basignin (Ok Blood Group, BSG), alanyl aminopeptidase (ANPEP), dendritic cell-specific intercellular adhesion molecule-3-grabbing non-integrin/cluster of differentiation 209 (DC-SIGN, CD209), C-type lectin domain family 4 member G/M (CLEC4G/M), and dipeptidyl peptidase-4 (DPP4) [52,53,54,55]. Several cellular proteases were also included, such as TMPRSS4 which functions with a priming factor; TMPRSS11A/B, which activates the S peptide of other coronaviruses; furin, which activates MERS-CoV and possibly SARS-CoV-2 proteins; and cathepsins (CTSL/B), which can substitute TMPRSS2 to prime SARS-CoV [56,57]. Additionally, there are restriction factors known to protect cells against SARS-CoV-2 entry such as lymphocyte antigen 6 family member E (LY6E) and interferon-induced transmembrane proteins 1, 2, and 3 (IFITM1–3) [57,58].

At the post-entry level, DNA topoisomerase iii beta (TOP3B) and zinc-finger CCHC-type and RNA-binding motif containing 1 (ZCRB1) are essential for SARS-CoV-2 and SARS-CoV genome replication, respectively [59,60]. Proteins involved in the assembly and trafficking of RNA viruses that physically interact with SARS-CoV-2 structural proteins include members of the Rho-GTPase complex (RHOA, RAB10, RAB14, and RAB1A), members of the activating protein 2 (AP2) complex (AP2A2 and AP2M1), and charged multivesicular body protein 2A (CHMP2A) [61].

More recently, SARS-CoV-2 was reported to form complexes with self-proteins to exploit receptor-mediated endocytosis through the interaction of its spike with soluble ACE2 (sACE2) or sACE2–vasopressin via angiotensin II receptor type 1 (AT1), the target of angiotensin receptor blockers (ARBs), or arginine vasopressin receptor 1B (AVPR1B), respectively [62]. Notably, sACE2 was at some point contemplated as a therapeutic agent for COVID-19, as it could compete for cell membrane ACE2 and, thus, decrease viral entry into cells [63,64,65]. However, the only results reported in clinicaltrials.gov were disappointing (NCT04335136). The fact that sACE2 can actually facilitate virus entry through alternative receptors may contribute to explaining the disappointing results. Additionally, neuropilin 1 (NRP-1) served as an entry factor that potentiates SARS-CoV-2 infectivity in vitro [66].

## 9. Susceptibility to SARS-CoV-2 Infection and Severity of COVID-19

Environmental, clinical (e.g., CKD), and social factors play a key role in SARS-CoV-2 infection and the severity of COVID-19, but host genetic factors may also contribute, as indicated above for resistance to clinical infection [6]. A full understanding of the biological role of genetic factors in the pathogenesis of COVID-19 may help to identify mechanistic targets for therapeutic development [67]. However, susceptibility to SARS-CoV-2 infection is not easy to define, due to the different viral, host, and environmental factors, in addition to diverse phenotypes, vaccination response, and population differences, among others. Different designs have been adopted to evaluate the role of genetics on the susceptibility and severity of COVID-19, as recently summarized [5]. Information is derived mainly from studies designed to identify common single nucleotide polymorphisms (SNPs) and rare and ultra-rare variants associated with different phenotypes, obtained from analyses of single genes or candidate-pathway association studies, genome-wide association studies (GWAS), meta-analyses, or polygenic risk scores [67,68,69,70,71,72,73,74,75,76,77,78].

## 10. SCARF Genes and Kidney Disease

CKD is considered a systemic condition. Thus, CKD modifies the physiology and function of multiple organs, inducing a state of systemic inflammation and accelerating biological aging [79,80]. This is the consequence of the retention of uremic toxins or the loss of key kidney functions, such as the clearance of small protein proinflammatory mediators or the production of the anti-inflammatory and anti-aging protein Klotho [81,82,83]. This altered pathophysiological state may be predicted to alter the expression of multiple genes, potentially including SCARF and COVID-19 susceptibility genes. Additionally, both AKI and CKD are associated with cause-specific and shared changes in kidney gene expression, many of which are the consequence of local inflammation. As an example, kidney diseases studied up to now share the early loss of Klotho expression, which is driven by tubular cell stressors, including inflammatory cytokines [84,85]. Finally, AKI, COVID-19, and SARS-CoV-2 infection of kidney cells result in specific local gene expression patterns (Table 1).

We have used two approaches to address the impact of kidney disease on the expression of genes relevant to COVID-19 susceptibility or severity either locally in the kidney or systemically.

In an experimental interventional approach followed by the validation of kidney data in human kidney transcriptomic databases, we induced CKD in mice through the addition of adenine to food and assessed the kidney and systemic expression of 21 SCARF genes [85]. Mice with adenine-induced CKD retain uremic solutes, and, thus, allow us to explore the systemic impact of kidney disease, unlike other models of CKD, like unilateral ureteral obstruction, that allow for the study of the local molecular mechanisms of kidney injury but not the systemic consequences given the fact that one kidney is normal [86].

In mice with adenine-induced CKD, 20/21 (95%) SCARF genes studied were differentially expressed in at least one organ [51,87,88,89,90,91,92,93] (Table 2). This very high rate of differential gene expression already suggests that expanding the analysis to other SCARF or disease susceptibility genes may uncover further abnormalities that sensitize to more severe COVID-19. Indeed, for 15/22 (68%) SCARF genes, the differential expression would be expected to favor SARS-CoV-2 infection and/or severity (Figure 4). The largest impact of CKD on COVID-19-related gene expression was observed in the kidneys. Of the 15 differentially expressed genes whose differential expression would be expected to favor SARS-CoV-2 infection and/or severity, 13 were differentially expressed in the kidney, and 8 of them were validated in human CKD kidney transcriptomic datasets, including those for the most common cause of CKD, diabetic nephropathy.

Two genes were reported to protect from SARS-CoV-2 and were downregulated in at least one non-kidney target organ: *Ifitm3* in the lung and *Ly6e* in the aorta [51]. This means that the downregulation of these genes during CKD may favor SARS-CoV-2 entry into lung or vascular cells, potentially increasing the severity of lung or vascular manifestations of COVID-19 in patients with CKD. The lungs and the vasculature are critical drivers of COVID-19 mortality. These findings should be validated in protein studies in the lungs or aorta of patients with CKD, ideally including immunohistochemistry studies that locate the cells where the differential expression is occurring. Additionally, the identification of the drivers of these changes in gene expression may provide tools to prevent or reverse them: Are they driven by uremic toxins, or by inflammatory cytokines or other factors? It may be argued that other SCARF genes in these organs are not differentially expressed in mice with CKD or even that the differential gene expression would be predicted to be protected from COVID-19. However, we should consider the context of this research: We already know that CKD patients are at increased risk of severe COVID-19, and the aim is to identify potential drivers of this increased risk. The differential expression of genes predicted to potentially result in protection from COVID-19 would not have a clinical translation, meaning that either they are not so influential on the overall pathophysiology of the disease, or they occur in non-relevant cell types, or they are not translated into functional protein changes.

The second approach was based on data extraction from the 237 SCARF genes and genes proposed to be involved in COVID-19 severity or SARS-CoV-2 susceptibility retrieved from the literature described above [5,53,54,55,57,59,60,62,67,75,77,94,95,96,97,98,99,100,101,102,103,104,105,106,107,108,109,110,111,112,113,114,115,116,117,118,119,120,121,122,123,124,125,126,127,128,129,130] (Supplementary Table S1). They were assessed in murine and human transcriptomic databases.

First, we evaluated the expression in a normal murine kidney database (Table 3). The rationale would be that early proof-of-concept studies will be more feasible in murine models, and there are already humanized murine models of coronavirus infection [131]. A total of 178 COVID-19-related genes were found in the normal kidney database, with 143 genes considered to be expressed. Additionally, we evaluated the expression of COVID-19-related genes in a human AKI single-cell transcriptomic database [132]. In bulk kidney data, 94 out of 225 found genes (41.8%) were differentially expressed during human AKI (Table 3): Of those, 22 were upregulated ≥1.25-fold, and 34 were downregulated to less than 0.5-fold. All the analyzed cell types had some degree of differential expression of COVID-19-related genes, with the bulk of them being differentially expressed in the thick ascending limb of Henle and proximal tubular cells, while podocytes were on the other side of the spectrum (Table 3), likely because they are not primary targets of the most common cause of AKI, formerly known as acute tubular necrosis.

Finally, differentially expressed genes in the kidneys of patients with COVID-19 and AKI included 19 of 237 COVID-19 susceptibility genes: *RPL24, OAS1-3, ARHGAP27, PPP1R15A, GOLGA3, XCR1, MX1, RUSC1, TCF19, POLD1, NOTCH4, HLA-E, PROC, GC, MRPS21, PDE4A, and ATP5PO* [35].

## 11. Proposal for a Research Agenda

**Figure 5:**
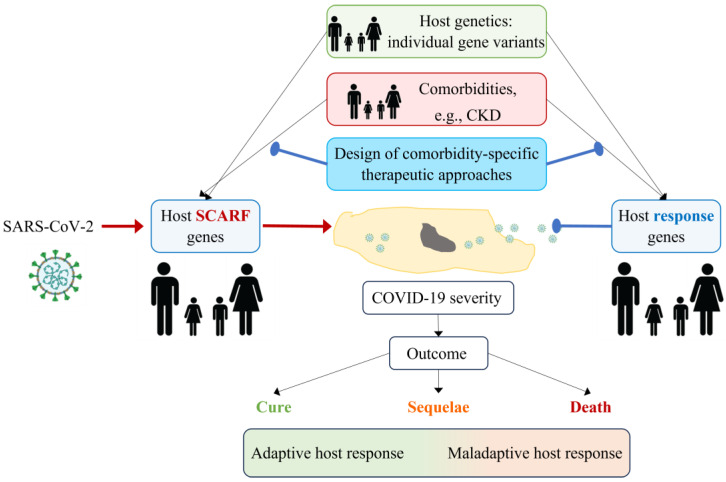
**Development of comorbidity-specific therapeutic approaches for COVID-19.** The severity of COVID-19 depends on the interaction between SARS-CoV-2 and the host. SARS-CoV-2 hijacks host gene products (SCARFs) to enter cells and replicate, while host response genes try to eliminate the virus. Genetic variants in the host may determine whether SCARFs and host response genes are present and whether they are expressed and active at appropriate levels. Additionally, comorbidities (e.g., CKD) may modify the expression or activity of SCARFs and host response genes or their products. Proof-of-concept studies have demonstrated that CKD modifies the systemic expression of some SCARF genes in a manner that may facilitate viral entry and replication and, potentially, increase COVID-19 severity. The characterization of such interactions between comorbidities, SCARFs, and host response genes may help design comorbidity-specific therapeutic approaches to SARS-CoV-2 or future viral threats that minimize the risk of sequelae or death. SCARF: coronavirus-associated receptors and factors, CKD: chronic kidney disease.

Overall, there is convincing evidence that kidney disease is associated with an increased risk of COVID-19 death. Indeed, CKD is the most common risk factor for severe CKD and the one that most increases the risk of COVID-19 death, while the development of AKI during a COVID-19 episode also increases the risk of death. On top of this, vaccination in patients with CKD in response to the disease may be suboptimal in terms of the duration of protective anti-spike protein antibodies or even, for kidney transplant recipients, in terms of the development of protective antibodies. In recent months, a better understanding of genetic risk factors for severe COVID-19 has inaugurated a new era, in which the impact of comorbidities on the expression and activity of these key genes may be explored to eventually develop comorbidity-specific therapeutic approaches (Figure 5). Given its well-characterized impact on multiple organs and systems, CKD is an ideal candidate for proof-of-concept studies that address this hypothesis. Early preclinical evidence backed by data extracted from human transcriptomic databases suggests that kidney injury is associated with the local differential expression of multiple genes involved in SARS-CoV-2 pathogenicity or the host response against it. Moreover, the differential gene expression was also observed in target organs of COVID-19 in mice with CKD. Some of these changes in gene expression would be expected to favor a higher severity of COVID-19. Box 1 summarizes potential next steps in the quest for comorbidity-specific approaches to fight SARS-CoV-2 or future pandemics caused by coronaviruses or other viruses. The research agenda proposes to advance preclinical research to initial clinical translation (the observational validation of key preclinical findings) to advanced clinical translation (interventional studies), recognizing that preclinical studies may not be feasible for certain genes whose expression levels and/or functions significantly differ between humans and mice. Preclinical studies should start from a data-mining exercise that identifies all human and murine datasets that are informative on SCARF and COVID-19 host gene expression in different organs under conditions of CKD. This should be followed by cross-validation between human and murine data and the characterization of the regulators of differentially expressed genes. Ideally, the function of key genes should be explored in humanized models of COVID-19 in mice, as this will facilitate advances in the functional analysis of specific genes through gene-targeting approaches. The key murine observational data then should be validated in human samples. Finally, samples from interventional human studies should be assessed to understand how key COVID-19 host genes are regulated in human CKD. Finally, if safe drug interventions are identified (i.e., drug repurposing), a prospective study should assess the feasibility of modulating COVID-19 host genes in order to increase the resistance of CKD patients to COVID-19, eventually testing whether this is protective. The analysis should not be limited to gene expression, as protein levels and even protein function may be modified in CKD, for example, through post-translational modifications such as carbamylation and others.

Box 1 **Toward developing comorbidity-specific therapeutic approaches. A research agenda.**

Preclinical proof of concept *

- Use data-mining strategies to assemble all the available information from public databases and explore the expression for all known COVID-19 host genes in the kidneys and key target organs (e.g., lungs, vasculature, etc.) of mice with CKD;
- Develop ad hoc models of murine CKD to fill the gaps in knowledge. This will facilitate the development of preclinical studies using tools such as genetically modified animals;
- Gene expression, protein levels, and protein post-translational modifications and function may be explored;
- Once potential targets have been identified that may account for the increased severity of COVID-19 in patients with CKD:
  o Characterize the CKD-related modifiers that modulate gene or protein expression or function;
  o Confirm the role of the specific target in the severity of COVID-19 in humanized mouse models.

(*Given the differences between humans and mice, for some genes, preclinical studies should be obviated as they will not be informative*).

**Initial clinical translation**

- Use data-mining strategies to explore the expression for all known COVID-19 host genes in the kidneys and key target organs (e.g., lungs, vasculature, etc.) of persons with CKD;
- Use focused analysis to confirm findings in murine models;
- Use focused biobank searches to address persistent gaps in knowledge.

**Advanced clinical translation**

- Identify interventions that restore the expression and function of the CKD-influenced COVID-19 genes that appear key for the increased severity of COVID-19;
- Initiate clinical development of such therapeutic strategies.

## Figures and Tables

**Table 1:** Top 10 upregulated (A) and downregulated (B) genes in human COVID-19 AKI that are not significantly differentially expressed (adjusted *p* value < 0.05) in human non-COVID-19 AKI when either of them is compared with control. Data obtained from ref. [35].

(A) Upregulated Genes			(B) Downregulated Genes		
Gene	Fold-Change *	*p*-Adj Value	Gene	Fold Change *	*p*-Adj Value
*FABP4*	36.0	0.002	*GC*	0.09	0.026
*APOBR*	23.5	0.000	*SLC22A24*	0.10	0.005
*S100A9*	19.4	0.050	*OR2T35*	0.11	0.012
*CHTF18*	13.0	0.004	*CRYM*	0.11	0.000
*KIFC1*	12.4	0.006	*GJA3*	0.11	0.023
*NFAM1*	11.9	0.006	*NAT8B*	0.11	0.008
*PSTPIP1*	11.9	0.000	*SLC6A18*	0.12	0.018
*BTNL9*	11.3	0.001	*RNU2-1*	0.12	0.018
*TRPM2*	10.8	0.000	*CYP4A22*	0.13	0.028
*FER1L4*	10.2	0.002	*AGT*	0.13	0.007

* Fold-change difference in gene expression of human AKI COVID-19 vs. control.

**Table 2:** SCARF genes that are differentially expressed in kidneys, lungs, heart, or aorta in mice with CKD [51].

Gene	Differential Gene Expression May Favor COVID-19 Severity				Kidney Differential Gene Expression Validated in Human CKD *
	Kidney	Lung	Heart	Aorta	
*Ace2*	No	No	No	No	Decreased
*Agtr1*	No	No	No	No	Increased
*Ap2a2*	Yes	No	No	No	1 Increased, 1 decreased
*Ap2m1*	Yes	No	No	No	Decreased
*Avpr1b*	No	No	No	No	Increased
*Cd147/Bsg*	No	No	No	No	No change
*Cd26/Dpp4*	Yes	No	No	No	Decreased
*Chmp2a*	Yes	No	No	No	Increased
*Nrp1*	Yes	No	No	No	Increased
*Nrp2*	Yes	No	No	No	Increased
*Rab10*	Yes	No	No	No	Increased
*Rab14*	Yes	No	No	No	Increased
*Rhoa*	Yes	No	No	No	Increased
*Tapt1*	Yes	No	No	No	Increased
*Tmprss2*	Yes	No	No	No	Decreased
*Tmprss4*	Yes	No	No	No	Increased
*Top3b*	Yes	No	No	No	Decreased
*Ifitm1*	No	No	No	No	Increased
*Ifitm2*	No	No	No	No	Increased
*Ifitm3*	No	Yes	Yes	No	Increased
*Ly6e*	No	Yes	Yes	Yes	Increased

* In red are those whose change in kidney gene expression is concordant for murine and human disease and their expression would be expected to increase the risk of severe COVID-19, and in green are those that are concordant, but their expression would not be expected to increase the risk. Supplementary Tables S1 and S2 (from reference [51]).

**Table 3:** Expression of 237 genes proposed to be involved in COVID-19 severity or SARS-CoV-2 susceptibility (Supplementary Table S1) in the murine normal kidneys [133] or human AKI kidneys [132]. Data are expressed as the number of genes or number (% of differentially expressed genes among genes expressed by that cell).

Database	Found	Significant Expression */Differential Expression **	Upregulated (≥1.5×) ***	Downregulated (≤0.5×) ***
Normal murine kidney	190	143	N/A	N/A
Human AKI (Bulk)	225	94 (42%)	19	34
Human AKI PT	140	50 (36%)	14	25
Human AKI podocyte	118	17 (14%)	3	11
Human AKI thin-limb Henle	106	35 (33%)	5	18
Human AKI TAL	128	58 (45%)	5	31
Human AKI DCT	94	27 (29%)	5	1
Human AKI CNT	113	32 (28%)	4	22
Human AKI CD-PC	107	20 (19%)	5	10
Human AKI CD-IC-AC	108	29 (27%)	4	18
Human AKI CD-IC-B	103	13 (13%)	2	9
Human AKI EC	95	15 (16%)	3	6
Human AKI IntC	83	13 (16%)	3	5
Human AKI leukocytes	88	7 (8%)	2	3

* Normal kidney; ** AKI vs. normal kidney, differentially expressed with *p* value < 0.05; *** AKI vs. normal kidney, *p* value < 0.05; N/A: Not applicable, PT: proximal tubules, TAL: thick ascending limb of Henle, DCT: distal convoluted tubule, CNT: connecting tubule, CD-PC: collecting duct principal cells, CD-IC-AC: collecting duct intercalated cells type A, CD-IC-B: collecting duct intercalated cells type B, EC: endothelial cells, IntC: interstitial cells.

## Supplementary Materials

The following supporting information can be downloaded at: https://www.mdpi.com/article/10.3390/ijms242216078/s1.
